# Supplementary material for: Risk Factors for Malnutrition among IBD Patients
Source: Nutrients. 2021 Nov 16;13(11):4098. doi: 10.3390/nu13114098 (PMC8622927; doi:10.3390/nu13114098)
Supplement: Supplementary file 1 [file nutrients-13-04098-s001.zip › nutrients-1398050-SI.pdf]

Table S1. Common malnutrition risk screening tools and their components

	NRS-2002[9]	MUST[6]	NRI[29]	MST[7]	MIRT[25]	SaksIBD-NR[26]
Target population	Severely ill patients who are undernourished		Peri-operative hospitalized patients	Hospital admitted patients	IBD patients	IBD patients
Validation against nutritional assessment tools	Clinical outcomes of disease	Fat free muscle index, Skeletal muscle index	Post-operative clinical outcome (complications, morbidity, mortality)	SGA	SGA<br>Clinical outcome of CD	MUST<br>RD/GI judgment of clinical nutritional status
**Parameters evaluated (points measured)**						
Weight parameters	**BMI**<br><18.5 + impaired general condition (3)<br>18.5-20 + impaired general condition (2)<br>20> (0)	**BMI (kg/m²)**<br><18.5 (2)<br>18.5-20 (1)<br>20> (0)	/	/	**BMI (kg/m²)**<br><18.5 (2)<br>18.5-20 (1)<br>20> (0)	/
	**Weight loss**<br>>5% in 1 months (3)<br>>5% in 2 months (2) >5% in 3 months (1)	**Weight loss (last 3-6 months)**<br>≥10% (2)<br>5-10% (1)<br><5% (0)	**Present weight/usual weight (6 months before illness)** *41.7	**Recent unintentional weight loss**<br>>15 kg (4)<br>11-15 kg (3)<br>6-10 kg (2)<br>1-5 kg (1)<br>Unsure (2)<br>No (0)	**Weight loss (last 3 months)**<br>≥10% (3)<br>5-10% (2)<br><5% (0)	**Weight loss (last month)**<br>>15 lbs (3)<br>10-15 lbs (2)<br>5-10 lbs (1)<br><5 lbs (0)
Dietary intake	**Reduced dietary intake (last week)**<br>Food intake 0-25% of normal requirement (3)<br>Food intake 25-50% of normal requirement (2)<br>Food intake 50-75% of normal requirement (1)	**Patient is acutely ill and there has been/likely to be no nutrition intake** > 5 d<br>Yes/no (2)	/	/	/	**Food restriction**<br>Yes/no (2)
Eating related symptoms	/	/	/	**Decreased appetite**<br>Yes/no (1)	/	**Symptoms (nausea/vomiting/diarrhea/poor appetite > 2 wk)**<br>≥3 symptoms (2)<br>1-2 symptoms (1)<br>No symptoms (0)<br>**Decreased appetite**<br>Yes/no (2)
Disease related parameters	**Disease severity:**<br>Absent – normal nutritional requirements (0)<br>Mild: hip fracture, chronic patients with acute complications such as COPD, cirrhosis, hemodialysis, diabetes, oncology (1)<br>Moderate: abdominal surgery, stroke, pneumonia, hematological malignancy (2)	/	**Serum albumin** (g/L)*15.9	/	**Serum CRP (mg/l)**<br>≥50 (3)<br>5-50 (2)<br><5 (0)	/

	NRS-2002[9]	MUST[6]	NRI[29]	MST[7]	MIRT[25]	SaksIBD-NR[26]
	Severe: head injury, bone marrow transplantation, ICU patients (3)					

Abbreviations: BMI: Body mass index; CRP: C-reactive protein; ICU: Intensive care unit. NST: Nutrition screening tools; NRS-2002: Nutrition Risk Screening 2002; MUST: Malnutrition universal screening tool; NRI: Nutritional Risk Index; MIRT: Malnutrition Inflammation Risk Tool; SaskIBD-NR: Saskatchewan Inflammatory Bowel Disease Nutrition Risk; RD/GI: Registered Dietitian and Gastroenterologist; MNA: Mini-Nutritional Assessment; FFMI: Fat Free Mas Index; SMI: Skeletal Muscle Index; SGA: Subjective Global Assessment; CRP: C- reactive protein; COPD: chronic obstructive pulmonary disease; ICU: intensive care unit.

Table S2. Demographic and clinical characteristics of the study population at the index visit

	Total population N=118	Controls n=59	Cases n=59	*p*
**CD Montreal phenotype (%) among CD patients, n=80**				
A1 - Below 16 years	20.0	18.9	21.6	0.937
A2 - Between 17 and 40 years	69.3	71.1	67.6	
A3 - Above 40 years	10.7	10.5	10.8	
L1 - Ileal	36.8	34.2	39.5	0.887
L2 - Colonic	19.7	21.6	18.4	
L3 - Ileo-colonic	43.4	44.7	42.1	
L4 – Proximal disease	18.4	18.9	18.4	1.000
B1 - Non-stricturing, non-penetrating	47.4	50.0	44.7	0.412
B2 - Stricturing	27.6	21.1	34.2	
B3 - Penetrating	25.0	28.9	21.1	
Perianal disease	34.2	26.3	42.1	0.147
**UC Montreal phenotype (%) among UC patients, n=26**				
E1 - Proctosigmoiditis	17.9	28.6	7.1	0.060
E2 - Left sided colitis	42.9	21.4	64.3	
E3 - Extensive colitis (extends beyond the flexure)	39.3	50.0	28.6	
S0 - remission (SCCAI ≤2)	28.6	35.7	21.4	0.523
S1- mild (SCCAI 3-5)	46.4	50.0	42.9	
S2 - moderate (SCCAI 6-9)	21.4	14.3	28.6	
S3 - severe (SCCAI ≥10)	3.6	0.0	7.1	
Age of diagnosis (years) (mean±std)	25.3±14.7	25.4±14.1	25.5±15.0	0.931
**Disease activity at baseline**				
**Physician global assessment (PGA) (%)**				
**Normal**	**32.2**	**47.5**	**16.9**	**<0.001**
**Mild**	**36.4**	**40.7**	**32.2**	
**Moderate**	**23.7**	**11.9**	**35.6**	
**Severe**	**7.6**	**0.0**	**15.3**	
**Abdominal pain (n=117) (%)**				
**None**	**59.0**	**69.5**	**48.3**	**0.008**
**Mild**	**19.7**	**16.9**	**22.4**	
**Moderate**	**13.7**	**13.6**	**13.8**	
**Severe**	**7.7**	**0.0**	**15.5**	
**Stool frequency (number/day)** (mean±std)	**4.1±4.4**	**3.1±3.2**	**5.0±5.3**	**0.026**
Blood in stool (n=114) (%)	14.9	10.5	19.3	0.189
Incontinence (n=113) (%)	10.6	6.9	14.5	0.187
Annual number of physician consultations/year (mean±std)	2.93±1.81	2.68±1.23	3.19±2.22	0.409
**Annual number of multi-disciplinary clinic visits/year** (mean±std)	**0.82±1.40**	**0.53±1.04**	**1.12±1.64**	**0.041**

Abbreviations: CD – Crohn's disease, UC – ulcerative colitis, SCCAI – simple clinical colitis assessment index, PGA – physician global assessment.

Table S3. Adjusted associations between disease and patient characteristics and malnutrition development.

	**Model 1**<br>**OR (95%CI),** ***p***	**Model 2**<br>**OR (95%CI),** ***p***
**18.5≤BMI≤22 kg/m²**	**15.14 (5.89-38.92)**<br>**<0.001**	**9.53 (3.20-28.37)**<br>**<0.001**
**Abdominal pain [a]**	**2.64 (1.03-6.77)**<br>**0.042**	**3.41 (1.03-11.27)**<br>**0.044**
High Stool frequency [b]	**2.47 (1.04-5.86)**<br>**0.039**	1.05 (0.36-3.06)<br>0.916
**Endoscopic disease activity [c]**	**4.87 (1.82-13.04)**<br>**0.002**	**7.30 (1.80-28.14)**<br>**0.004**
**High annual healthcare utility [d]**	**3.65 (1.48-9.02)**<br>**0.005**	**5.58 (1.79-17.40)**<br>**0.003**
Elimination diet	**2.51 (1.10-5.74)**<br>**0.028**	1.53 (0.53-4.39)<br>0.423
**CRP > 0.5 mg/dl**	**4.96 (2.09-11.76)**<br>**<0.001**	**9.15 (2.39-35.05)**<br>**0.001**
Calprotectin ≥ 200 μg/gr	**4.40 (1.01-19.06)**<br>**0.047**	5.95 (0.72-48.97)<br>0.097
**Steroid therapy**	**4.54 (1.38-14.94)**<br>**0.013**	**7.23 (1.75-29.84)**<br>**0.006**

Model 1: adjusted for disease duration and age of diagnosis. Model 2: adjusted for disease duration, age of diagnosis and MUST score at index visit. [a] Abdominal pain was defined as moderate/severe abdominal pain. [b] High stool frequency was defined as ≥5 defecations/day. [c] Endoscopic disease activity was defined as moderate/severe endoscopic disease. [d] High annual healthcare utility was defined as ≥5 physician/multidisciplinary clinic visits per year. Abbreviations: BMI – body mass index, CRP – C reactive protein.

Table S4. The association between the number of IBD-MRfactors and malnutrition preventive interventions

	**0 malnutrition risk factors (n=28)**	**1 malnutrition risk factors (n=47)**	**2 malnutrition risk factors (n=20)**	**3 malnutrition risk factors (n=15)**	***p***
Referral to a dietician (%)					
Controls	10.7	5.3	0.0	0.0	0.540
Cases	17.9	25.0	20.0	20.0	0.875
Partial enteral nutrition (%)					
Controls	0.0	5.3	0.0	0.0	0.381
Cases	10.7	33.3	6.7	14.5	0.106
Addition of advanced therapy (%)					
Controls	0.0	5.3	12.5	0.0	0.331
Cases	14.3	8.3	13.3	12.7	0.684

IBD-MR (range 0-3) was calculated as the sum of identified factors: BMI≤22kg/m^2, endoscopic disease activity, high annual health-care utility.
